# Histone modifications affect differential regulation of TGFβ- induced NADPH oxidase 4 (NOX4) by wild-type and mutant p53

**DOI:** 10.18632/oncotarget.17892

**Published:** 2017-05-16

**Authors:** Howard E. Boudreau, Wei Feng Ma, Agnieszka Korzeniowska, Jonathan J. Park, Medha A. Bhagwat, Thomas L. Leto

**Affiliations:** ^1^ Laboratory of Host Defenses, National Institute of Allergy and Infectious Diseases, National Institutes of Health, Bethesda, Maryland, USA; ^2^ Bioinformatics Support Program, National Institutes of Health Library, National Institutes of Health, Bethesda, Maryland, USA

**Keywords:** mutant p53, NOX4, p300, histone modification, cell migration

## Abstract

Previously, we showed wild-type (WT) and mutant (mut) p53 differentially regulate reactive oxygen species (ROS) generation by NADPH oxidase-4 (NOX4): p53-WT suppresses TGFβ-induced NOX4, ROS and cell migration, whereas tumor-associated mut-p53 proteins enhance NOX4 expression and cell migration. Here, we extended our findings on the effects of p53 on NOX4 in several tumors and examined the basis of NOX4 transcriptional regulation by p53 and SMAD3. Statistical analysis of expression data from primary tumors available from The Cancer Genome Atlas (TCGA) detected correlations between mut-p53 and increased NOX4 expression. Furthermore, by altering p53 levels in cell culture models we showed several common tumor-associated mutant forms support TGFβ/SMAD3-dependent NOX4 expression. Deletion analysis revealed two critical SMAD3 binding elements (SBE) required for mut-p53-dependent NOX4 induction, whereas p53-WT caused dose-dependent suppression of NOX4 transcription. ChIP analysis revealed SMAD3 and p53-WT or mut-p53 associate with SBEs and p53 response elements in a TGFβ-dependent manner. Interestingly, the repressive effects of p53-WT on NOX4 were relieved by mutation of its transactivation domain or histone deacetylase (HDAC) inhibitor treatment. Overexpression of p300, a transcriptional co-regulator and histone acetyltransferase (HAT), enhanced p53-mediated NOX4 induction, whereas HAT-inactive p300 reduced NOX4 expression. Mut-p53 augmented TGFβ-stimulated histone acetylation within the NOX4 promoter. Finally, wound assays demonstrated NOX4 and p300 promote TGFβ/mut-p53-mediated cell migration. Our studies provide new insight into TGFβ/SMAD3 and mut-p53-mediated NOX4 induction involving epigenetic control of NOX4 in tumor cell migration, suggesting NOX4 is a potential therapeutic target to combat tumor progression and metastasis.

## INTRODUCTION

The tumor suppressor gene *TP53* is the most commonly mutated gene in human cancers. Approximately 50% of all human cancers produce an inactive mutated tumor suppressor protein [1]. Tumor-associated p53 mutations are primarily missense mutations within the DNA binding domain that can give rise to a dominant-negative protein or a protein that has taken on a gain-of-function (GOF) with pro-oncogenic effects [1, 2]. Tumor-associated p53 mutants have been shown to support an increase in cell proliferation, invasion, migration, angiogenesis, resistance to chemotherapeutic drugs, and tumor development in animal models [3–7]. Several reports have demonstrated that expression of GOF p53 mutants in p53-*null* cell models results in up-regulation of genes associated with cell survival, proliferation, and migration, whereas genes involved in cell cycle arrest and apoptosis are down-regulated [8, 9]. However, the molecular mechanisms underlying gene expression changes that support the GOF phenotypes are still unclear.

Transforming growth factor-beta (TGFβ) is a pluripotent cytokine that can have either tumor-suppressing or tumor-promoting effects. As tumors progress, they respond to TGFβ by increasing their mobility and invasiveness, developing a more metastatic phenotype. Studies have shown that p53 plays an important role in TGFβ/SMAD2/3-mediated cell signaling and migration [10–12]. The switch between TGFβ being a tumor suppressor to a tumor promoter was shown to involve mut-p53 and SMAD2 forming a complex with p63 that inhibits p63 tumor suppressor function [11, 13]. Another recent study demonstrated an ERK-mediated interaction between mut-p53 (R175H) and SMAD3, but not SMAD2, regulates subsets of TGFβ target genes that promote cell migration and invasion [13]. Conversely, Cordenonsi *et al*. demonstrated p53-WT/SMAD2/3 complexes promote cell cycle arrest and apoptosis [14].

While studies have shown mut-p53 recognizes different regulatory sequences of its target genes, it is still unclear whether mut-p53 has a distinct DNA-binding consensus sequence. Several groups have reported on proteins that interact with mutant GOF p53, and found many of them are transcription factors and co-regulators, suggesting mut-p53 may indirectly regulate transcription through recruitment and complex formation [15]. Recent studies have provided evidence that GOF mut-p53 proteins bind to and increase the expression of chromatin regulatory genes such as methyltransferases and acetyltransferases, thereby increasing histone methylation and acetylation, and subsequently favoring a pro-oncogenic transcriptional program and phenotype [16, 17].

The transcriptional co-activator p300 is a histone acetyltransferase (HAT) that can acetylate histones and several transcription factors. A recent report suggested mut-p53 enhanced gene expression through recruitment and interaction with p300 and SMAD3 in many cancer cell types [17]. Contrary to this, they found that p53-WT associates with histone deacetylases (HDAC) in a co-repressor complex inhibiting gene expression [18, 19]. Another study showed phosphorylated p53-R175H binds to p300, strengthening transcriptional activity [20].

Previously, we showed wild-type and mutant forms of p53 differentially regulate reactive oxygen species (ROS) generation by NADPH oxidase 4 (NOX4) in breast and lung epithelial tumor lines [21]. We found that p53-WT suppresses TGFβ-induced NOX4, ROS production, and cell migration, whereas tumor-associated mut-p53 proteins (R175H and R280K) enhance NOX4 expression and cell migration by a TGFβ/SMAD3-dependent mechanism. The mechanisms underlying TGFβ/SMAD3/p53-based NOX4 regulation remain elusive. However, a recent report shed light on TGFβ-induced NOX4 gene expression by identifying a critical SMAD3 binding site in an upstream NOX4 transcriptional regulatory sequence [22]. Furthermore, histone modifications by acetlylation and methylation were recently shown to play an important role in epigenetic regulation of NOX4 gene expression [23].

Here, we examined the basis of NOX4 promoter regulation by p53 and SMAD3. Analysis of upstream NOX4 transcriptional regulatory sequences, identified critical SMAD3 binding elements (SBE) and p53 response elements (p53RE) required for mut-p53-induced NOX4 expression. Moreover, expression of active SMAD3 results in robust NOX4 promoter activity, which is abolished when co-expressed with p53-WT. The repressive effect by p53-WT on NOX4 is relieved upon treatment with HDAC inhibitors. Furthermore, overexpression of p300, a known mut-p53-binding transcriptional co-regulator with HAT activity, enhances mut-p53-mediated NOX4 promoter activity and cell migration. Together these results provide further insight on regulation and epigenetic control of NOX4 by TGFβ/SMAD3 and p53.

## RESULTS

### Differential regulation of TGFβ-induced NOX4 by wild-type and mutant p53

**Figure 1:**
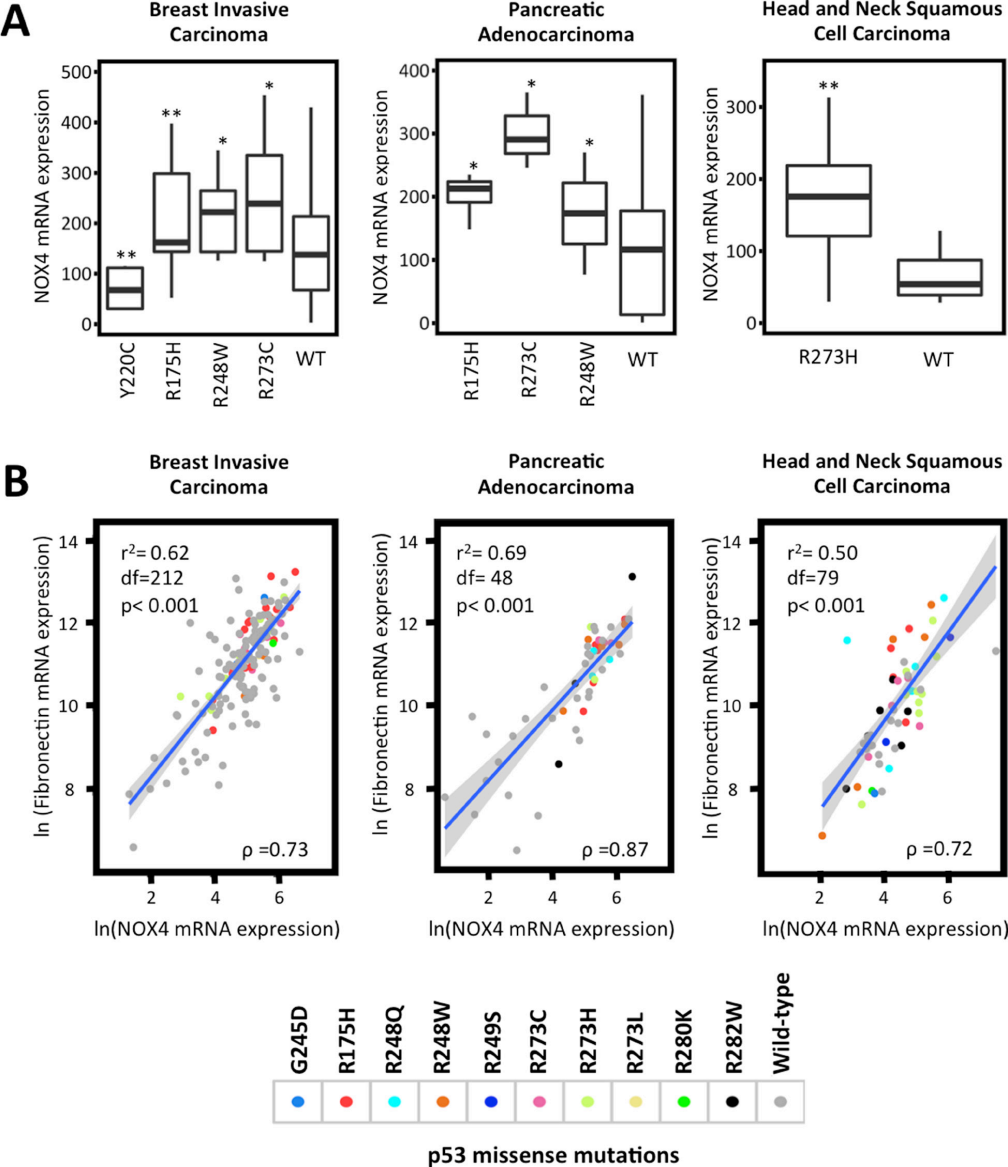
*TP53* mutation status correlates with NOX4 expression Clinical sample data from TCGA were extracted via cBioPortal. Samples were grouped by cancer studies and by p53 mutation status. (**A**) Breast invasive carcinoma (R175H [*n* = 42], R248W [*n* = 14], R273C [*n* = 12], Y220C [*n* = 11], wild-type [*n* = 116]), pancreatic adenocarcinoma (R175H [*n* = 5], R273C [*n* = 3], R248W [*n* = 5], wild-type [*n* = 29]) and head and neck squamous cell carcinoma (R273H [*n* = 16] and wild-type [*n* = 17]) NOX4 mRNA expression in samples with different p53 mutations were compared to those with p53-WT within their respective cancer studies. Statistical significance was established using Mann-Whitney test where appropriate. Extreme outliers were removed from plots but retained during statistical analysis. (**B**) Increases in NOX4 mRNA expression are positively correlated with fibronectin expression patterns. TCGA data were analyzed using Spearman rank correlation tests and linear regression analysis. Data points were colored based on the assigned p53 mutation status. The R script, copies of the data used and high-resolution figures are publically available at github.com/wfma/HEBoudreau. Significance values are indicated as *P-value < 0.05, or **P-value < 0.01.

To elucidate the mechanisms involved in p53 regulation of NOX4, we first sought to confirm whether p53 mutation status is correlated with NOX4 mRNA expression in different primary tumor samples. We performed statistical analysis on gene expression data available from TCGA. Here we found a correlation between commonly occurring p53 missense mutations within the “hot-spot” DNA binding domain region and increased expression of NOX4 in several tumor types. Breast invasive carcinoma samples with p53 mutations exhibited higher NOX4 expression relative to tissues with p53-WT (Figure 1A). However, tumor samples with mut-p53-Y220C correlated with reduced NOX4 expression indicating that not all DNA binding domain p53 mutants result in higher NOX4 expression. Further, p53 mutations in pancreatic adenocarcinoma and head and neck squamous cell carcinoma also were correlated with increased NOX4 expression relative to their WT counterparts (Figure 1A).

TGFβ is a well-known inducer of the epithelial-to-mesenchymal transition (EMT) transcriptional program involving SMAD3 activation, whereby genes involved in cell-cell junctions and epithelial polarization are down-regulated while genes associated with the extracellular matrix and migration such as fibronectin are up-regulated [24]. Previously, we examined TGFβ-induced fibronectin expression in relation to NOX4 and found p53-WT suppressed TGFβ induction of fibronectin, similar to NOX4 [21]. Here, we sought further evidence supporting the correlation between NOX4 and fibronectin expression patterns in relation to p53 mutation status in tumor samples from TCGA. Interestingly, increased NOX4 and fibronectin mRNA expression positively correlated in tissue samples with p53 DNA binding domain ‘hot-spot” mutations (Figure 1B). Collectively, these data suggest mut-p53-induced NOX4 has a role in cancer progression in several tumor types.

**Figure 2:**
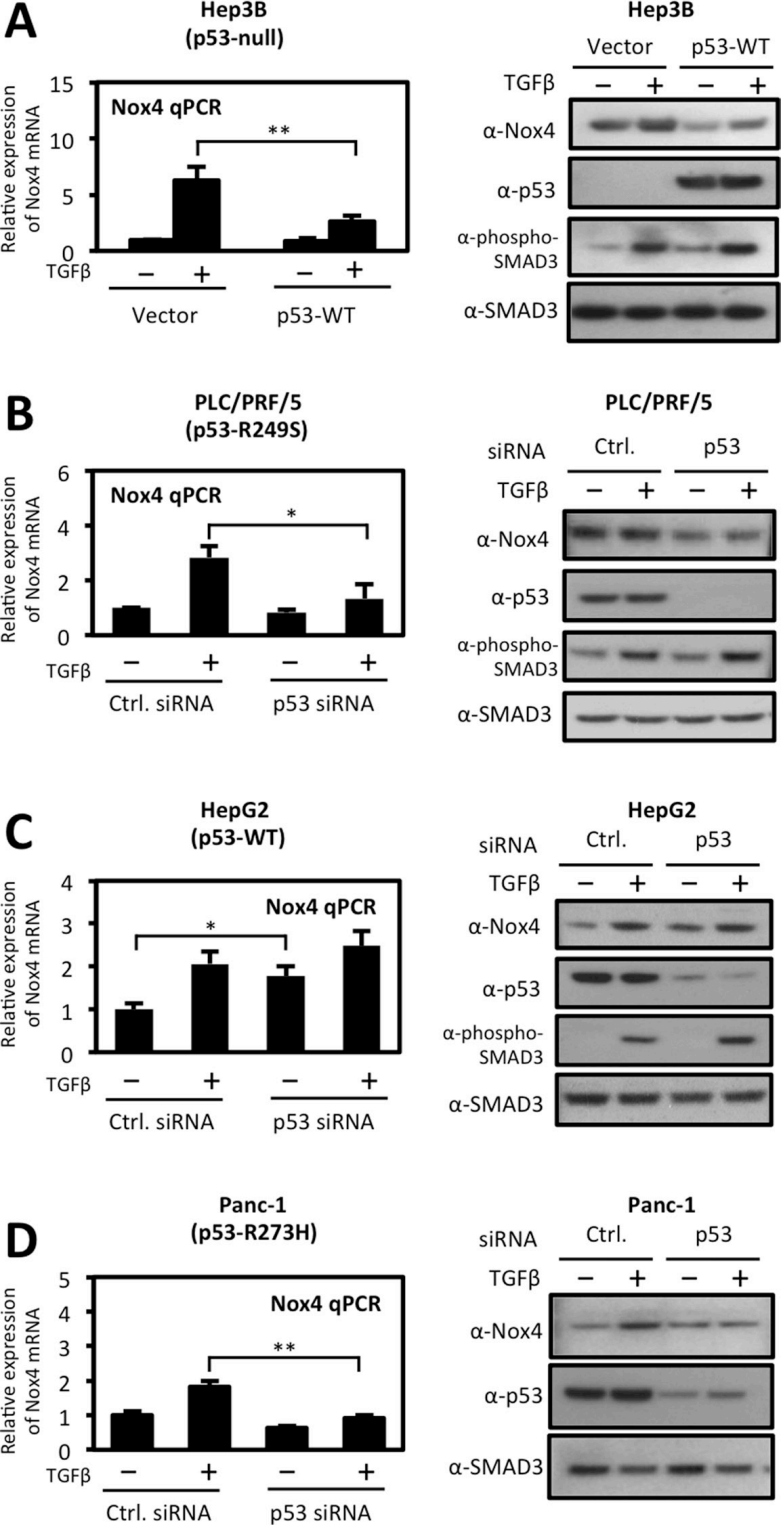
Differential regulation of TGFβ-induced NOX4 by wild-type and mutant p53 (**A**) *Left panel*, p53-*null* Hep3B cells were transfected with vector control or p53-WT plasmids. Twenty-four hours after transfection, cells were treated with TGFβ (5 ng/ml) for 24 hours. Real-time quantitative PCR (qPCR) analysis of mRNA expression was determined using human NOX4-specific primers. GAPDH-specific primers were used for a reference gene for normalization. Quantification of NOX4 mRNA is described relative to vector untreated cells. *Right panel*, Hep3B cells were transfected and treated as the left panel. Thirty micrograms of total cell lysates were analyzed by western blotting. The immunoblot was probed sequentially with antibodies against NOX4, p53, phospho-SMAD3, and total SMAD3. (**B**, **C** and **D**) Effects of siRNA-mediated silencing of endogenous wild-type or mut-p53 on NOX4 expression. (B) PLC/PRF/5 ^(p53-R249S)^, (C) HepG2 ^(p53-WT)^, and (D) PANC-1 ^(p53-R273H)^ cells were transfected with On-Target SMARTpool p53-specific siRNAs (50 nM) or non-targeting control siRNAs (50 nM) or for 48 h followed by TGFβ (5 ng/ml) treatment for an additional 24 h. Quantitative PCR of NOX4 mRNA (*left panel*) and protein expression (*right panel*) were analyzed as in panel A (*n* = 3). Significance values are indicated as *P-value < 0.05, or **P-value < 0.01.

We then explored the divergent effects of wild-type and mut-p53 on NOX4 expression in several established tumor cell models. We transfected p53-*null* Hep3B hepatocytes with p53-WT followed by TGFβ stimulation for 24 hours. Expression of p53-WT significantly reduced TGFβ-induced NOX4 at both the mRNA and protein levels (Figure 2A). Next, we transfected PLC/PRF/5 hepatocytes with p53-specific siRNAs to deplete these cells of the endogenously expressed p53-R249S. Knockdown of p53-R249S significantly reduced both TGFβ-induced NOX4 mRNA and protein expression (Figure 2B). We further confirmed our results in HepG2 cells, which express endogenous p53-WT. Depletion of p53-WT in HepG2 cells enhanced NOX4 expression in the presence or absence of TGFβ (Figure 2C). We also observed a decrease in TGFβ-induced NOX4 expression in PANC-1 pancreatic cancer cells upon silencing of endogenous p53-R273H (Figure 2D). Together, these results confirm that p53-WT suppresses whereas mut-p53 enhances TGFβ-mediated NOX4 expression in both hepatic and pancreatic tumor models.

### Several tumor-associated p53 mutants increase NOX4 mRNA and promoter activity in a TGFβ-dependent manner

**Figure 3:**
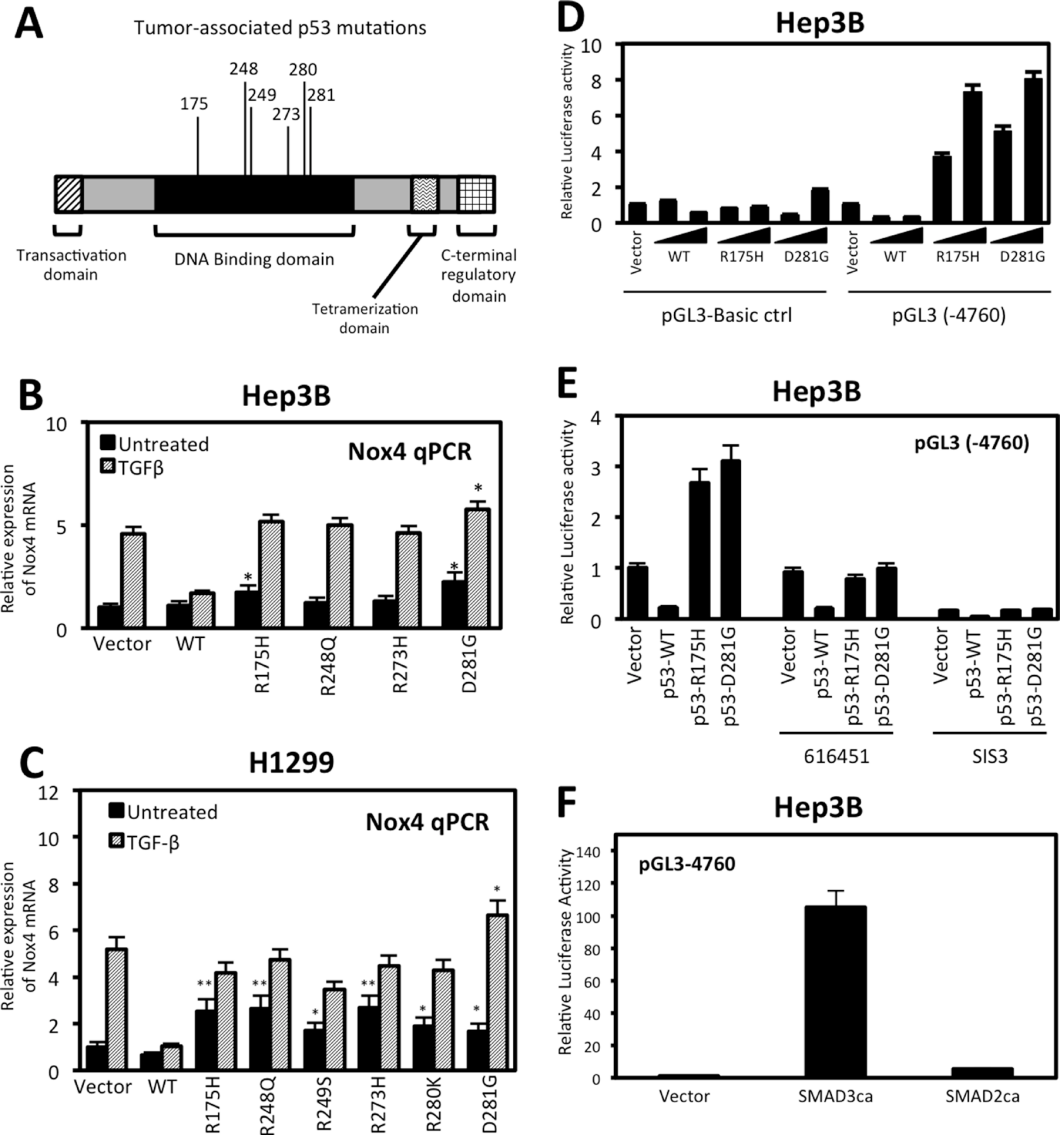
Tumor-associated p53 mutants increase NOX4 mRNA and promoter activity in a TGFβ-dependent manner (**A**) Schematic representation of the p53 functional domains showing common tumor-associated “hotspot” missense mutations within the DNA binding domain. (**B**) Hep3B cells were transfected with control, p53-WT, p53-R175H, p53-R248Q, p53-R273H or p53-D281G plasmids. Twenty-four hours after transfection, the cells were treated with TGFβ (5 ng/ml) or left untreated for an additional 24 hours. NOX4 mRNA expression was determined by qPCR analysis using NOX4 and GAPDH-specific primers. Results for NOX4 mRNA are quantified relative to vector untreated control (*n* = 3, in triplicate). (**C**) H1299 cells were transfected with vector control plasmid, p53-WT, p53-R175H, p53-R248Q, p53-R249S, p53-R273H, p53-R280K, or p53-D281G mutant plasmids. The cells were then treated and analyzed for NOX4 mRNA expression as in panel B (*n* = 3, in triplicate). (**D**) Hep3B cells were co-transfected with NOX4 promoter luciferase reporter plasmid pGL3-NOX4 (-4760) or pGL3-Basic control (0.5 μg) and increasing amounts (0.25 or 0.5 μg) of vector plasmids or p53-WT, p53- R175H, or p53-D281G plasmids for 48 hours. Total cell lysates were collected and assayed for luciferase activity by luminescence. Luciferase activity is described as fold change in relative light units compared to the vector untreated cells (*n* = 3, in triplicate). (**E**) Hep3B cells were co-transfected with NOX4 promoter luciferase reporter plasmid pGL3-NOX4 (-4760) (0.5 μg) and vector control plasmids or p53-WT, p53- R175H, or p53-D281G plasmids. After 24 hours, the cells were treated with 616451 (10 *μ*M), a TGFβR1-specific inhibitor or SIS3 (10 *μ*M), a SMAD3-specific inhibitor for 4 h before treating with TGFβ for 20 hours. Cell lysates were collected and assayed for luciferase activity by luminescence (*n* = 3, in triplicate). (**F**) Hep3B cells were co-transfected with NOX4 promoter luciferase reporter plasmid and control plasmid, SMAD3 constitutively active (SMAD3ca), or SMAD2 constitutively active (SMAD2ca) plasmids. Forty-eight hours post-transfection, cell lysates where analyzed for luciferase activity (*n* = 3, in triplicate). Significance values are indicated as *P-value < 0.05, or **P-value < 0.01.

We have previously shown that p53-R175H and p53-R280K enhanced TGFβ-induced NOX4 expression; here we examined other common tumor-associated p53 mutants on NOX4 gene expression (Figure 3A). First, we investigated NOX4 mRNA expression in response to TGFβ in Hep3B (p53-*null*) cells heterologously expressing p53-WT, p53-R175H, p53-R248Q, p53-R273H, p53-D281G or control plasmids (Figure 3B). Control cells and p53 mutant expressing cells treated with TGFβ for 24 hours displayed robust increases in NOX4 mRNA whereas expression of p53-WT diminished this effect. Interestingly, p53-R175H and p53-D281G mutants increased NOX4 mRNA in the absence of TGFβ stimulation. Furthermore, p53-D281G augmented TGFβ-induced NOX4 expression compared to treated control cells. These effects were confirmed in H1299 (p53-*null*) lung epithelial cells. H1299 cells expressing p53-WT, p53-R175H, p53-R248Q, p53-R249S, p53-R273H, p53-R280K, p53-D281G or control vectors were treated with TGFβ for 24 hours. NOX4 mRNA expression was substantially up-regulated in cells expressing mut-p53 compared to p53-WT with or without TGFβ stimulation (Figure 3C). Similar to the Hep3B hepatocytes, p53-D281G caused the greatest enhancement of NOX4 expression upon TGFβ treatment compared to control.

Next, we examined the effect of p53-WT and mutant expression on the NOX4 promoter. A sequence of 4,760 base pairs upstream from the NOX4 transcriptional start site cloned into a luciferase reporter plasmid (pGL3) [22] was used to measure promoter activity. Hep3B cells co-transfected with the NOX4 promoter-reporter and increasing concentrations of mut-p53 plasmids (p53-R175H, p53-D281G) displayed a dose-dependent increase in NOX4 promoter activity in comparison to pGL3-Basic empty control transfected cells (Figure 3D). These results show mut-p53 proteins increased NOX4 promoter activity even in the absence of TGFβ. However, we found mut-p53-mediated NOX4 promoter activity was TGFβ-dependent, since NOX4 promoter (-4760) activity induced by p53-R175H or p53-D281G was abolished with a TGFβR1-specific inhibitor, 616451, or a SMAD3 specific inhibitor, SIS3 (Figure 3E). To further validate the role of TGFβ/SMAD3 signaling on NOX4 promoter activation, we co-transfected the NOX4 promoter (-4760) with constitutively active forms of SMAD2 or SMAD3. A significant increase in promoter activity was observed in cells expressing constitutively active SMAD3, whereas active SMAD2 had minimal effect on the NOX4 promoter (Figure 3F). Collectively, these data demonstrate that several of the common tumor-associated mut-p53 proteins positively regulate NOX4 at both the mRNA and promoter level in a TGFβ/SMAD3-dependent manner, whereas p53-WT acts as a potent repressor of TGFβ-mediated NOX4 expression.

### Wild-type p53 is a potent repressor of TGFβ/SMAD3-induced NOX4 promoter activity

**Figure 4:**
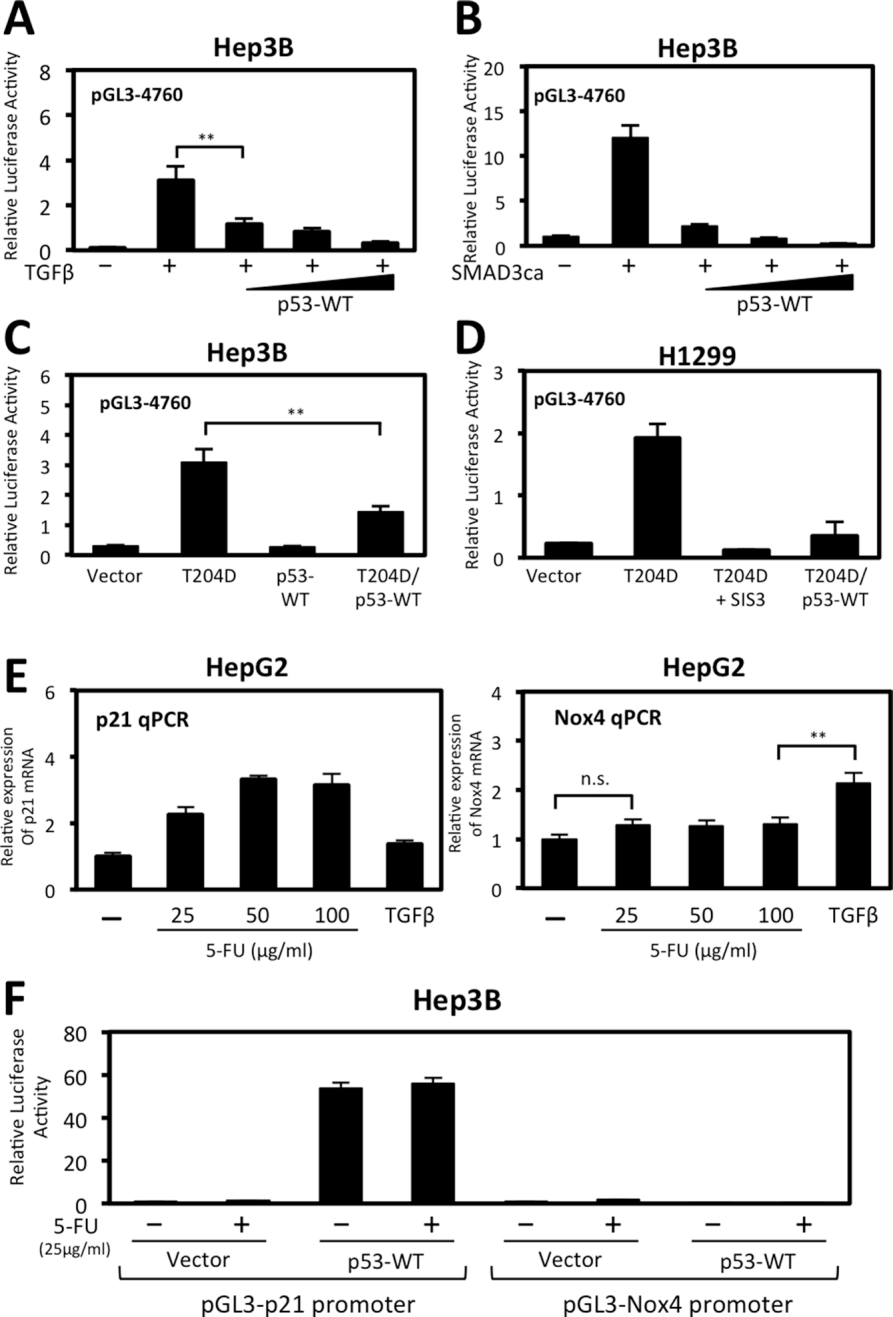
Wild-type p53 represses TGFβ/SMAD3-induced NOX4 promoter activity (**A**) Hep3B cells were co-transfected with NOX4 promoter-luciferase reporter plasmid pGL3-NOX4 (-4760) (0.5 μg) and vector control (0.5 μg) or increasing amounts of p53-WT (0.1, 0.3, or 0.5 μg) for 24 hours then either treated with TGFβ (5 ng/ml) for 24 hours or left untreated. Total cell lysates were collected 48 hours post-transfection and assayed for luciferase activity by luminescence (*n* = 4, in triplicate). (**B**) Hep3B cells were co-transfected with NOX4 promoter reporter and vector control or constitutively active SMAD3 (0.5 μg). Wild-type p53 was co-transfected at increasing concentrations as in panel A. Forty-eight hours after transfection, total cell lysates were assayed as in panel A (*n* = 3, in triplicate). (**C**) Hep3B cells were co-transfected with pGL3-NOX4 (-4760) and either vector control, constitutively active TGFβR1 (T204D), p53-WT, or T204D and p53-WT. After 48 hours, total cell lysates were collected for luciferase activity (*n* = 3, in triplicate). (**D**) H1299 cells transfected with the pGL3-NOX4 (-4760) promoter-reporter were co-transfected with either vector control, T204D, or with T204D and p53-WT. Twenty-four hours after transfection, SIS3 (10 *μ*M) was added for an additional 24 hours. Cell lysates were collected and assayed for luciferase activity. (**E**) HepG2 cells were treated with increasing concentrations of 5-fluorouracile (5-FU) (25, 50, or 100 μg/ml) or TGFβ (5 ng/ml) for 24 hours. Real-time quantitative PCR (qPCR) analysis of p21/CDKN1A mRNA expression (*right panel*) or NOX4 mRNA (*left panel*) was determined using gene-specific primers for human p21 or NOX4. Results are described as p21 or NOX4 mRNA expression relative to untreated control and normalized to GAPDH (*n* = 3, in triplicate). (**F**) Hep3B cells were co-transfected with p21 promoter reporter or pGL3-NOX4 (-4760) plasmids and control vector or p53-WT plasmids for 24 hours followed by 5-FU (25 μg/ml) treatment for an additional 24 hours. Total cell lysates were collected and assayed for luciferase activity (*n* = 3, in triplicate). Significance values are indicated as **P-value < 0.01 or n.s. (not significant).

We further explored the repressive effect p53-WT has on NOX4 promoter function and found p53-WT diminished TGFβ activation of the NOX4 promoter in a dose-dependent manner (Figure 4A). Similarly, when co-transfected with constitutively active SMAD3, NOX4 promoter activity (−4760) was diminished by increasing p53-WT expression (Figure 4B). Moreover, NOX4 promoter activation in cells expressing constitutively active TGFβR1 (T204D) was significantly blunted when co-expressed with p53-WT in Hep3B (Figure 4C) and in H1299 (Figure 4D) p53-*null* cell models.

Next, we compared the expression of NOX4 to p21/CDKN1A, a well-established activated p53 target gene involved in cell cycle arrest. We treated HepG2 cells which express endogenous p53-WT with 5-Fluorouracil (5-FU), a chemotherapeutic drug that induces p53-dependent cell growth arrest and apoptosis [25]. We found that p21 gene expression was induced in a 5-FU dose-dependent manner, but not in cells treated with TGFβ (Figure 4E, left). Conversely, NOX4 expression was induced by TGFβ but not by 5-FU treatments (Figure 4E, right). Moreover, in Hep3B cells co-transfected with p53-WT and a p21 promoter reporter plasmid, p21 promoter activity was significantly increased with or without 5-FU, whereas p53-WT and 5-FU has no effect on the NOX4 promoter (Figure 4F). Taken together, these data demonstrate p53-WT is a negative regulator of TGFβ-induced NOX4 promoter activity and that NOX4 is not a target gene responsive to p53-WT activation by 5-FU.

### The NOX4 promoter contains unique TGFβ/SMAD3 and p53 regulatory sequences

**Figure 5:**
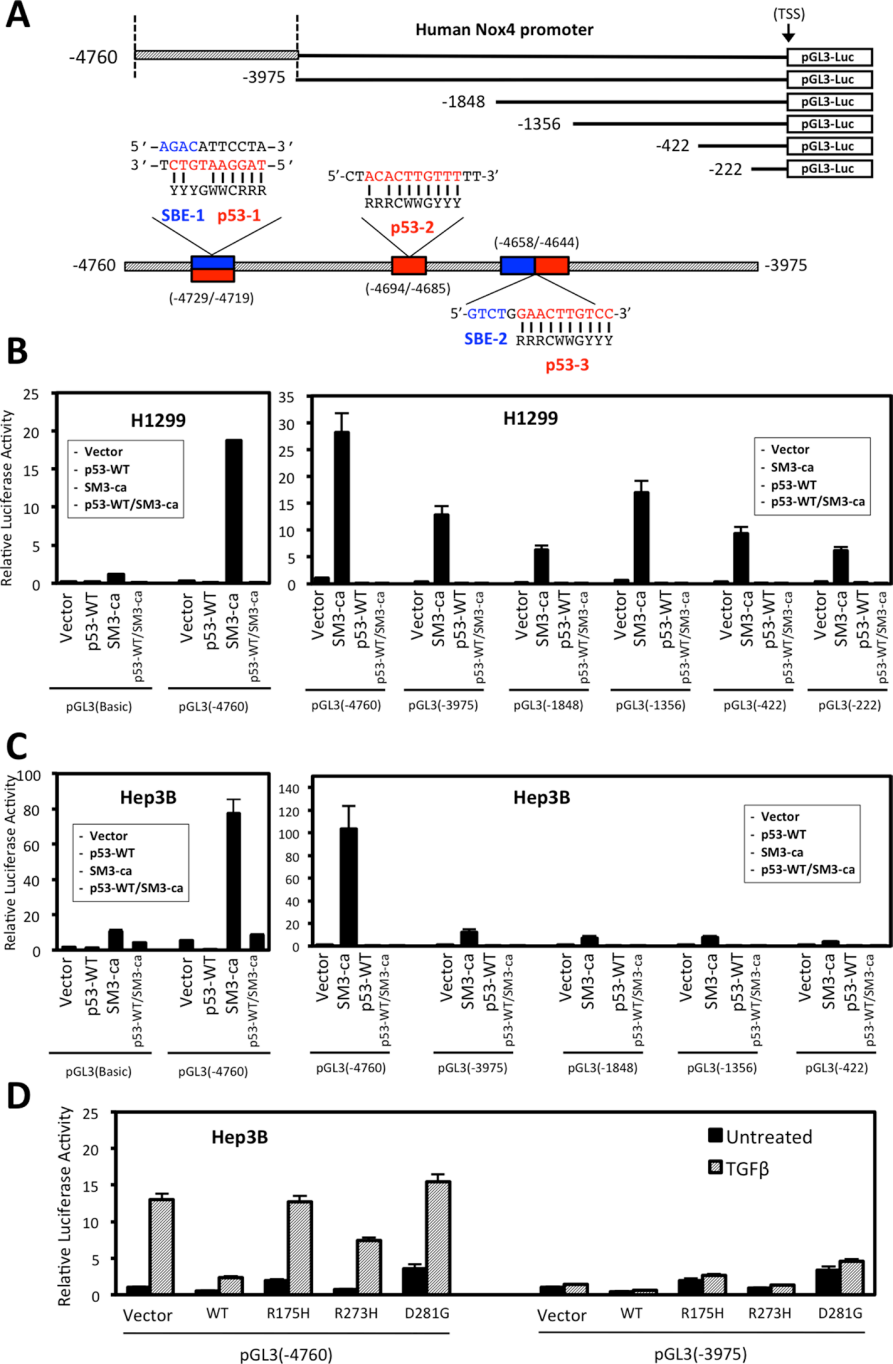
Deletion analysis of the NOX4 promoter reveals TGFβ/SMAD3 and p53 regulatory sequences (**A**) Schematic of 5′-end deletion series of the human NOX4 promoter starting from the transcription start site (TSS) was cloned into the pGL3-luciferase reporter vector to measure promoter activity. Putative SMAD binding elements (SBE) and p53 response elements are indicated within the -4760/-3975 promoter regions. The traditional p53 response element (p53-RE) consensus sequence consists of two 10-base decamers with a 0-13-base spacer: RRRCWWGYYY…*n* = 0-13 bp…RRRCWWGYYY (R is a purine (A/G), Y is a pyrimidine (C/T), and W is (A/T) [27]. However, studies have shown that p53 target genes that have one of the two 10-base decamers, or “half site” within the promoter can still be regulated by p53 binding [26, 27]. The consensus sequences for SMAD binding elements (SBE) are CAGA and its reverse complement GTCT.[41] Above SBE are indicated in blue and p53-RE in red. (**B**) H1299 cells and (**C**) Hep3B cells were co-transfected with designated pGL3-Basic control or NOX4 promoter reporter plasmids and either vector control, SMAD3ca, p53-WT, or both p53-WT/SMAD3ca plasmids. Luciferase activity was determined 48 hours after transfection (*n* = 3, in triplicate). (**D**) Hep3B cells co-transfected with NOX4 promoter pGL3 (-4760) or pGL3 (-3975) and either vector control, p53-WT, p53-R175H, p53-R273H, or p53-D281G plasmids for 24 hours. The cells were then left untreated or treated with TGFβ (5 ng/ml) for an additional 24 hours. Total cell lysates were collected following TGFβ treatment and analyzed for luciferase activity (*n* = 3, in triplicate).

Previous studies have indicated p53 and SMAD3 proteins can form a complex that regulates TGFβ-responsive genes [11]. Moreover, recent studies by Bai *et al*. identified a TGFβ/SMAD-responsive region of the NOX4 gene located between -3976 bp and -4760 bp upstream of the transcription start site and an AP-1/SMAD binding site located at -4667 bp to -4653 bp critical for TGFβ-mediated NOX4 promoter activity [22]. These findings prompted us to search for p53 response elements (RE) in close proximity to consensus SMAD binding elements (SBE). Several reports have shown that both wild-type and mutant p53 can mediate gene transcription using half-site response elements [26, 27]. We identified sequences that conform to consensus sequence for three half-site p53-REs and an additional SBE within the -3976 bp and -4760 bp promoter region of NOX4 (Figure 5A).

We analyzed a series of NOX4 deletion constructs to determine p53 and SMAD3 responsive regions (Figure 5A). Co-transfection of constitutively active SMAD3 and pGL3-NOX4 (-4760) resulted in robust promoter activity that was decreased in cells expressing the truncated pGL3-NOX4 (-3975) reporter construct (Figure 5B, 5C). SMAD3-induced promoter activity was completely diminished when co-transfected with p53-WT. Moreover, mut-p53 (p53-R175H and p53-D281G) expression supported TGFβ-stimulated (-4760) promoter activity. However, this activity was lost with co-transfection of the (-3975) reporter construct (Figure 5D). These data indicate p53-WT is a potent negative regulator of TGFβ/SMAD3-mediated transcription of NOX4, whereas mut-p53 supports NOX4 expression predominately through a TGFβ-dependent mechanism.

### Deletion of conserved SMAD binding elements or p53 response elements reduce NOX4 promoter activity induced by TGFβ

**Figure 6:**
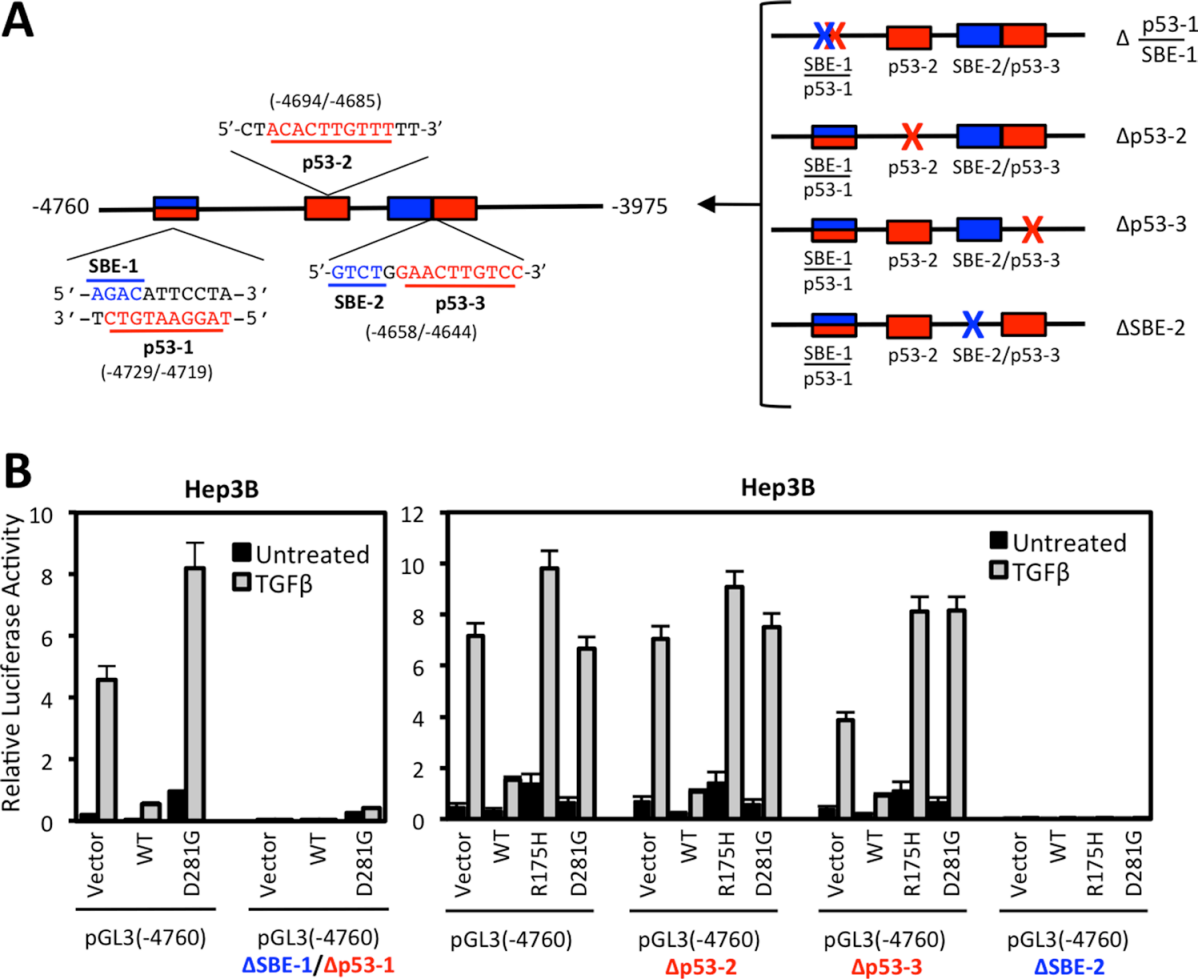
Deletion of conserved SMAD binding elements (SBE) or p53 response elements (p53-RE) reduce NOX4 induction by TGFβ (**A**) Schematic of the SBE (blue) and p53 response element (red) sequences and their position within the (-4760) to (-3975) segment of the NOX4 promoter (*left panel*). SBE (blue X) or p53RE (red X) sequences were deleted in the pGL3-NOX4 promoter-reporter (-4760) to determine functionality (*right panel*). (**B**) Hep3B cells were co-transfected with designated pGL3-NOX4 promoter-reporter vectors and either vector control, p53-WT, p53-R175H, or p53-D281G plasmids for 24 hours. Transfected cells were either left untreated or treated with TGFβ (5 ng/ml) for an additional 24 hours. Promoter activity was determined by luciferase assay (*n* = 3, in triplicate).

To explore contributions of putative SMAD binding and p53 response elements, we generated pGL3-NOX4 (-4760) promoter reporter constructs with deletions of either p53-1/SBE-1 overlapping sequences, p53-RE-2, p53-RE-3, or SBE-2 sequences (Figure 6A). Deletion of either the overlapping p53-1/SBE-1 or SBE-2 sequences completely abolished NOX4 promoter activity, whereas deleting p53-RE-2 or p53-RE-3 resulted in minimal changes in promoter activity (Figure 6B). Therefore, the overlapping SBE-1/p53-RE-1 and SBE-2 sequences are critical for TGFβ-mediated promoter activity.

### SMAD3 and p53 associate with NOX4 SBE and p53-RE sequences in a TGFβ-dependent manner

**Figure 7:**
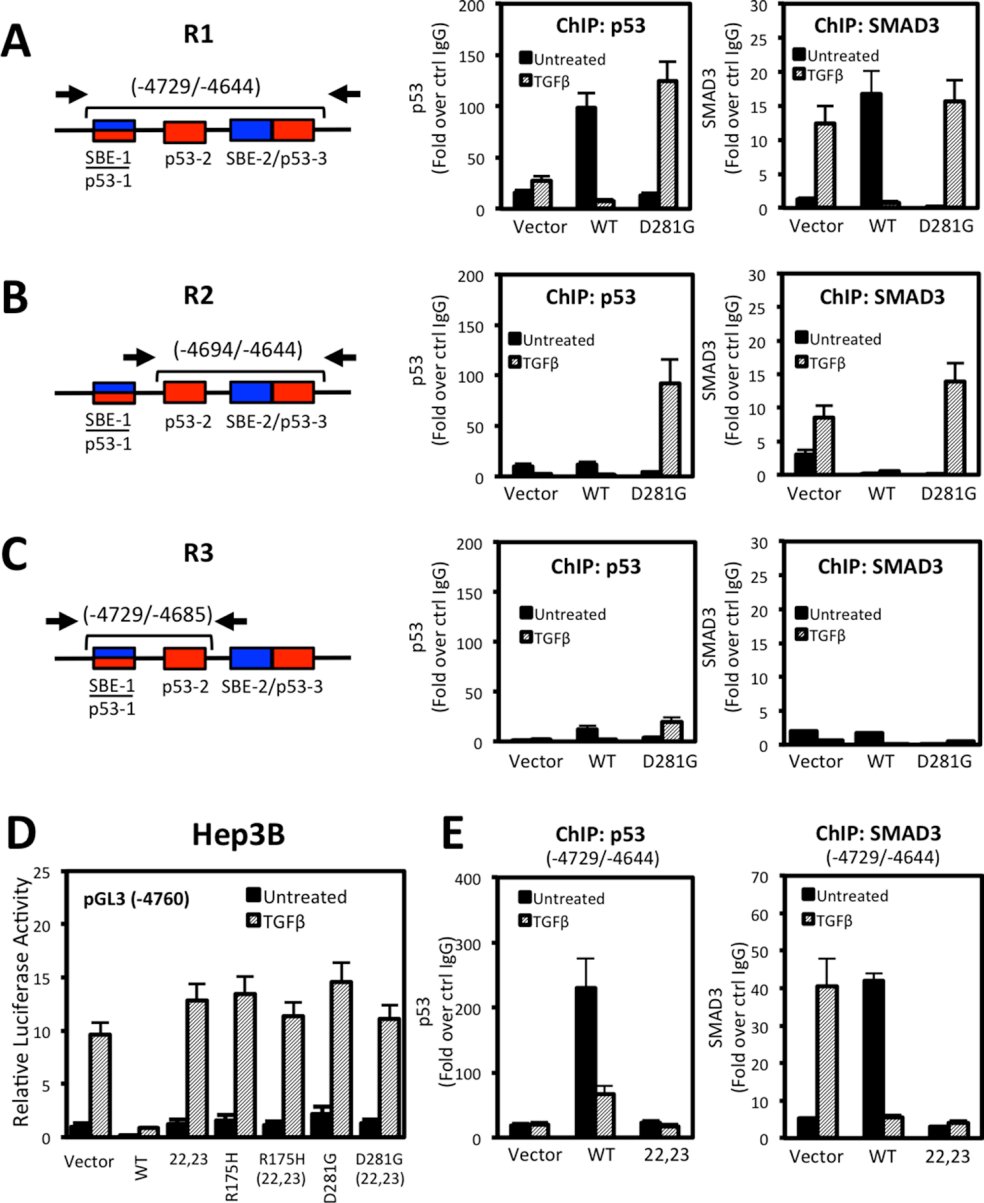
p53 and SMAD3 associate with NOX4 SBE and p53RE sequences in a TGFβ-dependent manner (**A**–**C**) Schematic showing the NOX4 promoter and the p53 and SMAD target regions tested in ChIP assays (*left*). See Materials and Methods for primer sequences. ChIP was performed in Hep3B cells transfected with vector control, p53-WT, or p53-D281G plasmids for 24 hours and subsequently treated with TGFβ (5 ng/ml) for an additional 24 hours. ChIP assays were performed using antibodies that specifically recognize p53, SMAD3, or IgG (negative control). Input DNA and immunoprecipitated DNA were quantified by qPCR. The ChIP-qPCR results are represented as fold enrichment of IP with anti-p53 (*middle*) or anti-SMAD3 (*right*) ChIP over input DNA relative to IgG negative control (*n* = 3). (**D**) Luciferase assays were performed in Hep3B cells using the pGL3-NOX4 promoter-reporter (-4760). The cells were transfected with control vector, p53-WT, p53-L22Q/W23S (transactivation domain mutant), mut-p53-R175H, p53-D281G, or the triple mut-p53-R175H/L22Q/W23S or p53-D281G/L22Q/W23S. Twenty-four hours after transfection, cells were left untreated or treated with TGFβ (5 ng/ml) for an additional 24 hours. (**E**) ChIP assay was performed as described in panel A in Hep3B cells that were expressing control, p53-WT, or p53-L22Q/W23S followed by 24 hours stimulation with TGFβ (5 ng/ml) (*n* = 2, in duplicate).

Next, we address molecular mechanisms involved in p53 and SMAD3 regulation of NOX4. We used p53 and SMAD3 specific antibodies to perform chromatin immunoprecipitation (ChIP) assays in Hep3B cells transfected with p53-WT, p53-D281G, or control vector followed by 24 hours TGFβ stimulation. We found that p53-WT and SMAD3 were recruited to NOX4 sequences in the absence of TGFβ, whereas p53-D281G and SMAD3 were recruited only in the presence of TGFβ (Figure 7A–7C). Further, SBE-1/p53-1 and SBE-2/p53-3 response elements were required for p53-WT and SMAD3 recruitment in the absence of TGFβ, whereas SBE-2/p53-3 elements were necessary for p53-D281G and SMAD3 recruitment in response to TGFβ.

Next, we examined whether the p53 transactivation domain of either wild-type or mutant was critical in regulating NOX4. Mutation of hydrophobic amino acids (L22Q/W23S) within the transactivation domain of p53 abolish co-factor binding and transactivation of target genes [28, 29]. Surprisingly, expression of the p53-L22Q/W23S double mutant was unable to repress TGFβ-mediated NOX4 promoter activity, suggesting the transactivation abilities of p53-WT are required for NOX4 repression (Figure 7D). Conversely, mutating the transactivation domain of the tumor-associated mutants (p53-R175H and p53-D281G) did not affect NOX4 promoter activity. To determine if a functional p53-WT transactivation domain is required for binding the NOX4 promoter, we preformed ChIP assays and found that unlike p53-WT, p53-L22Q/W23S did not precipitate with the -4729/-4644 region (Figure 7E). We also observed a significant loss of SMAD3 recruitment in cells overexpressing p53-L22Q/W23S, suggesting p53-WT and SMAD3 form a repressor complex inhibiting NOX4 expression.

### Histone deacetylase (HDAC) activity is involved in p53-WT-mediated repression of NOX4 mRNA expression and promoter activity

**Figure 8:**
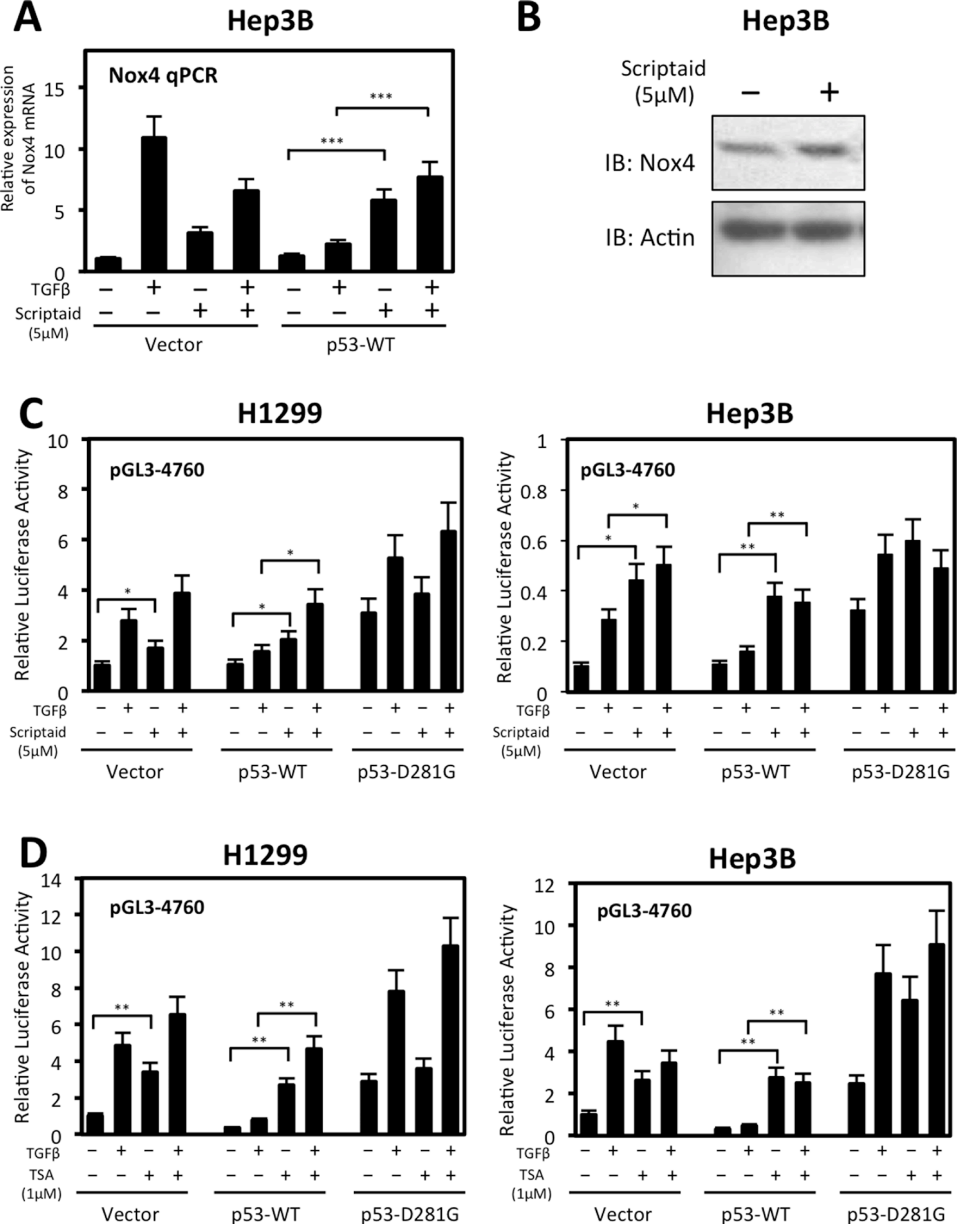
Histone deacetylase (HDAC) activity participates in wild-type p53-mediated repression of NOX4 mRNA and promoter activity (**A**) Hep3B cells were transiently transfected with control vector or p53-WT. Twenty-four hours after transfection, cells were treated with TGFβ (5 ng/ml) and either Scriptaid (5 μM) or DMSO for 24 hours. Real-time qPCR analysis of NOX4 mRNA expression was detected with NOX4-specific primers. The relative mRNA level was normalized to GAPDH control. (*n* = 4) (**B**) Hep3B cells transfected with empty vector were treated with Scriptaid (5 μM) for 24 hours. After 24 hours, total cell lysates were collected and analyzed by immunoblot. (**C**) H1299 or Hep3B cells were co-transfected with pGL3-NOX4 (-4760) and either vector control, p53-WT, or p53-D281G. Twenty-four hours later, cells were left untreated or treated with TGFβ (5 ng/ml) and either Scriptaid (5 μM) or DMSO (-) for another 24 hours. Total cell lysates were then collected and assayed for luciferase activity. (**D**) Luciferase assays were conducted on H1299 or Hep3B cells co-transfected with pGL3-NOX4 (-4760) p53 plasmids as in B. Twenty-four hours later, cells were left untreated or treated with TGFβ (5 ng/ml) and either trichostatin A (TSA) (1 μM) or DMSO (-) for 24 hours (*n* = 3, in triplicate). Significance values are indicated as *P-value < 0.05, **P-value < 0.01, or ***P-value < 0.001.

Previous studies demonstrated p53-WT can recruit HDACs to deacetylate histones associated with the promoters of genes involved in cell survival and migration [18, 30]. HDACs catalyze removal of acetyl groups from histone lysine residues, thereby inhibiting transcription factor binding and gene expression [31]. We examined the possibility of HDACs participating in p53-WT-based repression of NOX4. We found NOX4 mRNA expression was relieved of p53-WT repression in Hep3B cells treated with Scriptaid, an HDAC inhibitor, for 24 hours compared to untreated cells (Figure 8A). Interestingly, we observed an increase in NOX4 mRNA expression in the vector-transfected cells treated with Scriptaid, indictaing inhibition of HDACs also enhances basal expression of NOX4. Moreover, we observed a slight increase in NOX4 protein expression in response to HDAC inhibition indicating HDACs regulate NOX4 primarily at the transcriptional level resulting in increased NOX4 protein levels (Figure 8B). Furthermore, p53-WT repression of NOX4 promoter activity was lost upon treatment of H1299 or Hep3B cells with either Scriptaid or trichostatin A (TSA) HDAC inhibitors (Figure 8C, 8D). H1299 and Hep3B cells transfected with NOX4 promoter reporter treated with HDAC inhibitors relieved significant amount of basal promoter repression, indicating that histone deacetylation has a role in repressing NOX4 even in the absence of p53-WT.

### p300 histone acetyltransferase (HAT) activity enhances mutant-p53-mediated NOX4 promoter activity and cell migration

**Figure 9:**
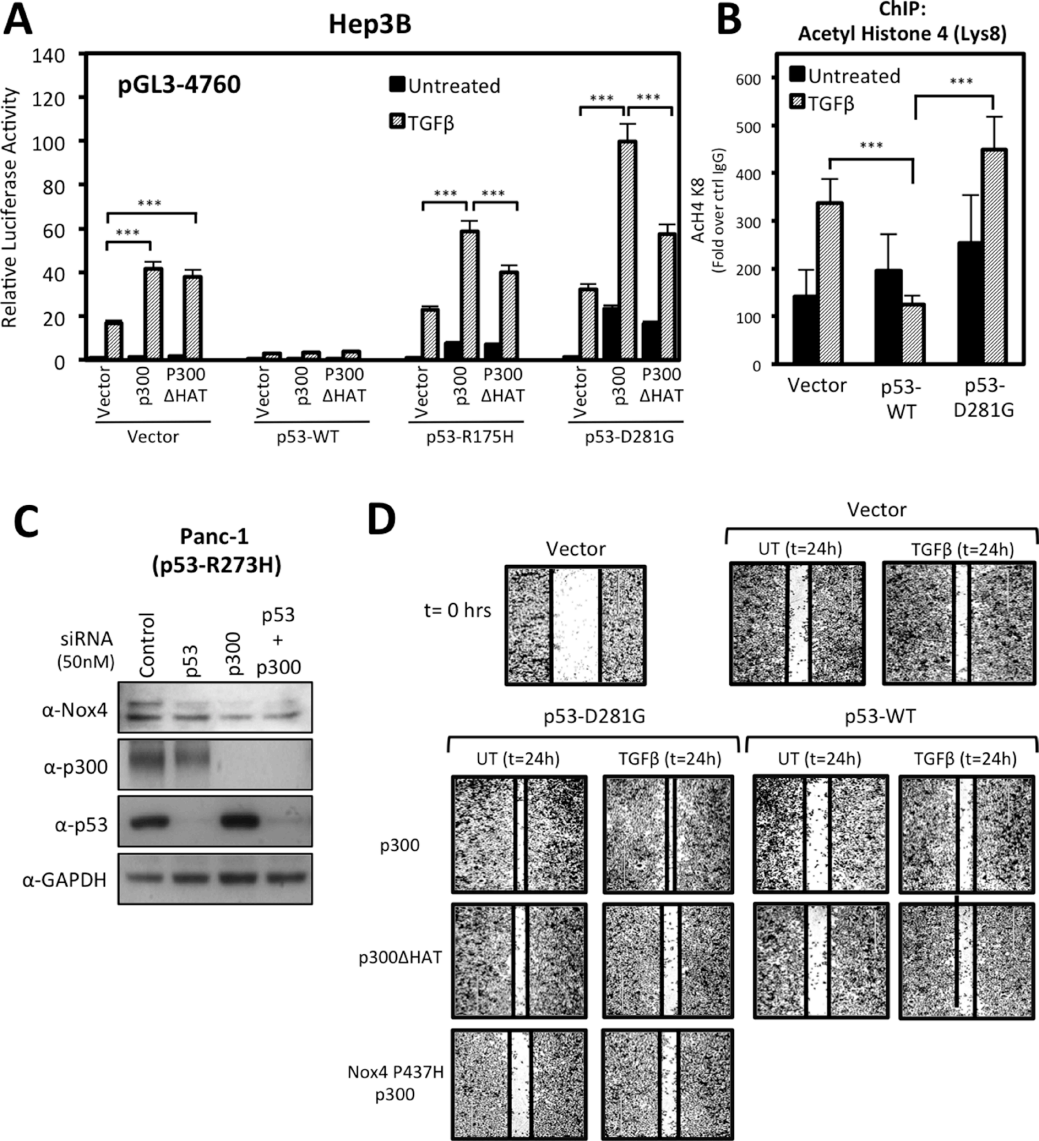
p300 histone acetyltransferase (HAT) activity enhances mutant p53-mediated NOX4 promoter activity and cell migration (**A**) Hep3B cells co-transfected with NOX4 promoter pGL3 (-4760) and either vector control, p53-WT, p53-R175H, or p53-D281G; and either control, p300, or HAT-inactive p300ΔHAT plasmids for 24 hours. The cells were then treated with TGFβ (5 ng/ml) for an additional 24 hours. Total cell lysates were collected following TGFβ treatment and analyzed for luciferase activity (*n* = 3, in triplicate). (**B**) ChIP assays were performed in Hep3B cells expressing control, p53-WT, or p53-D281G plasmids and either treated with TGFβ (5 ng/ml) for 24 hours or remained untreated. Histone immunoprecipitation was conducted using ChIP qualified antibodies specific for acetylated lysine-8 histone 4 (H4K8) antibodies, or IgG (negative control). ChIP-qPCR results are represented as fold enrichment of IP: α-H4K8 ChIP over input DNA relative to IgG negative control (*n* = 2, in triplicate). (**C**) PANC-1 ^(p53-R273H)^ cells were transfected with On-Target SMARTpool p53-specific siRNAs (50 nM), p300-specific siRNAs (50 nM), or non-targeting control siRNAs (50 nM) for 72 h. Fifty micrograms of total cell lysates were analyzed by immunoblotting. (**D**) H1299 cells transfected with vector control, p53-WT, p53-D281G, p300-WT, p300-ΔHAT, or NOX4-P437H plasmids and seeded into a 96-well tissue culture plate (3.5 × 10^4^ per well) and grown to confluence. Wounds were made using the WoundMaker 96-pin tool, which creates 96 precise and reproducible wounds of 800μm. The cells were then left untreated or were treated with TGFβ (5 ng/ml). Approximately 18-22 hours after wounding and TGFβ treatment, the cells were fixed and stained for imaging. The images presented are representative of four experiments completed in triplicate. Significance values are indicated as ***P-value < 0.001.

Next, we investigated the possibility of histone acetyltransferase (HAT) involvement in p53-mediated NOX4 expression. Studies have indicated tumor-associated p53 mutants affect histone modifications and transcriptional co-activation of multiple pro-oncogenic and pro-migratory genes [17, 20]. Moreover, mut-p53 was shown to interact with p300, a transcriptional co-activator and histone acetyltransferase, and promote the transcription of cell cycle regulatory genes [20]. When we co-transfected Hep3B cells with p300 and the NOX4 (-4760) promoter reporter, we observed a significant gain in TGFβ-induced promoter activity in control cells and in cells expressing p53-R175H or p53-D281G, but not p53-WT (Figure 9A). Conversely, co-expression of the HAT-inactive p300 mutant (p300ΔHAT) with p53-R175H or p53-D281G resulted in reduction of TGFβ-induced promoter activity relative to p300 co-expression, indicating HAT activity is involved in mut-p53-mediated NOX4 promoter activation. Interestingly, we found that HAT activity was not involved in TGFβ-induced promoter activity in control cells, suggesting p300 HAT activity is utilized specifically in mut-p53-dependent regulation of the NOX4 promoter.

Next, we examined the status of promoter histone acetylation by ChIP assay using an antibody specific for acetylated histone-4 lysine-8 (H4K8). Previous reports have shown that p300-mediated acetylation of H4K8 is associated with transcriptional activation [32]. We found TGFβ treatment increased H4K8 acetylation associated with the NOX4 promoter which was augmented by p53-D281G expression (Figure 9B). Conversely, p53-WT expression severely blunted H4K8 acetylation, suggesting histone modifications play a significant role in the divergent effects of mut-p53 and p53-WT on TGFβ-mediated NOX4 expression.

Thus far, our results indicate p300 and mutant p53 co-regulate the NOX4 promoter. These findings prompted us to investigate whether p300 and mutant p53 also have roles in basal NOX4 expression. To do this, we depleted the endogenous expression of p53-R273H, p300, or both by siRNA-mediated knockdown in PANC-1 pancreatic tumor cells. Knock down of either p53-R273H or p300 resulted in a reduction of basal NOX4 protein expression compared to control (Figure 9C). Collectively, these observations indicate p300 related epigenetic mechanisms are involved in mutant p53-dependent induction of Nox4.

We demonstrated in previous work that mut-p53 and NOX4 are necessary for TGFβ-mediated cell signaling and migration of different human tumor cell lines [21]. A recent study demonstrated p300 involvement in pancreatic tumor cell migration and invasion [33]. Therefore, we investigated whether p300 is involved in mut-p53/NOX4-dependent cell migration. We conducted scratch wound repair assays in 96-well plates using the 96-pin WoundMaker instrument to create 96 reproducibly uniform wounds approximately 800 μm in width. Wound assays conducted on H1299 cells demonstrated that co-expression of p53-D281G and p300 increased TGFβ-induced wound closure (Figure 9C). However, this increase in cell migration was reduced in cells co-transfected with dominant negative NOX4-P437H, confirming that NOX4 has a significant role in TGFβ-stimulated, p53-D281G/p300-mediated cell migration. Furthermore, we found that p300 HAT activity is critical in p300/p53-D281G-mediated wound closure, which corroborates our findings in Figure 8A showing that p300 with HAT activity supports TGFβ/mut-p53 induction of NOX4 promoter activity. In contrast, p53-WT alone or co-expressed with p300 resulted in a reduction in TGFβ-dependent wound closure.

## DISCUSSION

Previously, we described NOX4 as a TGFβ/SMAD3-inducible source of ROS in both normal and metastatic epithelial cells [34, 35]. We provided strong evidence of NOX4 having an important role in common EMT-related events including elevated cellular ROS production, increased fibronectin expression, and increased cell migration and invasion [34]. We also reported that the mutational status of p53 is an important determinant of TGFβ/SMAD3-mediated NOX4 regulation in breast and lung epithelial cells, showing that p53- WT is a suppressor of NOX4 induction whereas tumor-associated mutant forms of p53 enhance NOX4 expression and ROS generation [21]. Consequently, mut-p53-induced NOX4 results in increased focal adhesion kinase (FAK) activation and cell motility [21]. Here, we confirmed and extended these findings on differential regulation of NOX4 by mutant and p53-WT based on primary tumor expression data from TCGA and examined the effects of several common tumor-associated p53 mutants on NOX4 mRNA expression and promoter activity as well as tumor cell migration.

Our study provides insight into the mechanisms of p53 and SMAD3 transcriptional regulation of NOX4. We identified critical SBE and p53RE required for NOX4 induction by mut-p53 within the -4760/-3975 bp region upstream of the transcription start site of NOX4. Previously, Bai *et al*. identified an AP-1/SMAD box critical for transcriptional activation of NOX4 by TGFβ in lung fibroblasts [22]. This finding corroborates with our studies by deletion analysis of SBE-2 and by ChIP binding assays using SMAD3-specific antibodies. ChIP analysis of TGFβ-treated Hep3B cells revealed SMAD3 and p53-D281G are associated with the NOX4 promoter, whereas SMAD3 and p53-WT associate only in the absence of TGFβ. Furthermore, disruption of the transactivation domain of p53-WT (L22Q/W23S) caused a loss of its repressive effect on TGFβ-induced NOX4 promoter activity. In support of this, ChIP assays revealed a substantial loss of both SMAD3 and p53-L22Q/W23S binding to the NOX4 promoter, suggesting the transactivation domain is a required for SMAD3 associations with p53-WT as a negative co-regulator. However, it is worth noting that the distance of mut-p53 and SMAD3 binding (-4729/4644 bp) from the transcription start site suggests these factors are associated with an enhancer-like region or complex to regulate NOX4 transcription.

One mode of transcriptional repression by p53 is the recruitment of co-repressors and chromatin-modifying enzymes such as HDACs [36]. Histone acetylation is balanced by the activities of acetyltransferases and deacetylases. Acetyltransferases are associated with increased gene transcription and deacetylases with repressed gene expression [37]. Here, we show inhibition of HDACs abolished p53-WT repression of NOX4 promoter activity and mRNA expression, suggesting HDACs play a role in p53-WT-mediated NOX4 repression. Conversely, overexpression of p300 enhanced mut-p53-mediated NOX4 promoter activity, whereas the HAT-inactive p300 reduces these effects. Moreover, we observed a TGFβ-induced increase in acetylaytion of histones associated with the NOX4 promoter, which was augmented by p53-D281G. We also showed expression of an inactive mutant from of NOX4-P437H attenuated p53-D281G/p300-mediated cell migration stimulated by TGFβ. Here we provided further support for the involvement of p300 as a mediator of NOX4-dependent cell migration. The acetylation of histones associated with the NOX4 promoter by p300 may be an important epigenetic marker in determining the progression of tumors expressing mut-p53.

**Figure 10:**
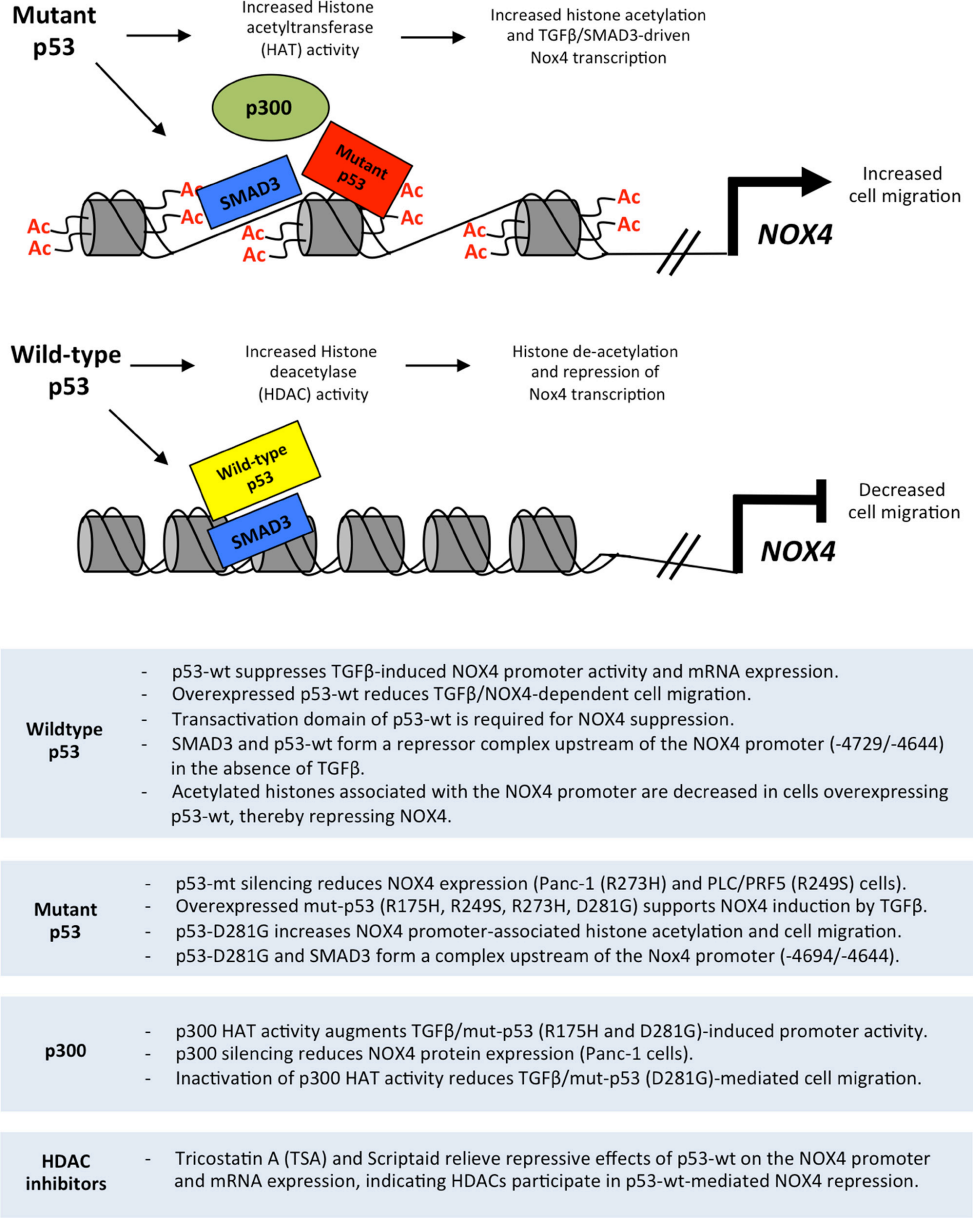
Model of the divergent roles of wild-type and mutant p53 regulating NOX4 expression and cell migration through histone modifications Mut-p53 and SMAD3 co-localize to SBE and p53-RE sequences in the NOX4 gene. Mutant p53 supports p300-mediated acetylation of histones associated with NOX4 regulatory sequences, which enhances TGFβ/SMAD3-driven NOX4 promoter activity and subsequent NOX4-dependent migration of tumor cells. In contrast, p53-WT and SMAD3 co-localize to SBE and p53-RE sequences to suppress NOX4. Histone deacetylases are recruited by p53-WT to the NOX4 locus, thereby repressing NOX4. Summary (below) outlines experimental evidence from the current study supporting the schematic model.

Together, our data suggest mut-p53 enhances TGFβ/SMAD3-driven NOX4 promoter activity involving histone modifications mediated by p300. In contrast, p53-WT requires a functional transactivation domain to exert its repressive effect on NOX4, suggesting p53-WT associates with other repressive co-factors. Transcriptional repression of NOX4 by p53-WT is also in part due to HDAC enzyme activity (Figure 10). Further investigation is necessary to establish additional co-factors involved in transcriptional regulation of NOX4 by both p53-WT and mut-p53. Our previous and current findings confirm a correlation between tumor-associated p53 mutations and increased NOX4 expression and downstream events promoting tumor cell migration, suggesting NOX4 is an attractive therapeutic target to abolish or decrease metastatic progression in multiple cancer types.

## MATERIALS AND METHODS

### The cancer genome atlas (TCGA) analysis

To identify whether p53 mutation status correlates with NOX4 mRNA expression, we extracted expression data as z-score values and mutation status from TCGA via cBioPortal [38, 39]. We performed Mann-Whitney Wilcoxon tests where appropriate to determine significant differences in NOX4 expression levels between mut-p53 and p53-WT. We also screened for possible relationship between NOX4 and fibronectin mRNA expression. We extracted fibronectin data from TCGA and employed Spearman rank correlation analysis as well as simple linear regression of the log_e_-transformed data to detect relationships between NOX4 and fibronectin mRNA expression. Statistical analyses were performed in R [40]. Samples with extreme outliers were not included in final plots but were included in the statistical analysis. The R script and copies of data used are available at github.com/wfma/HEBoudreau.

### Cell culture

Human cell lines H1299, PCL/PRF/5, HepG2, PANC-1, and Hep3B were from ATCC (Manassas, VA, USA). H1299 cells were maintained in RPMI (Thermo Fisher, Waltham, MA, USA) with 10% fetal bovine serum (FBS) (Thermo Fisher) and 100 mg/ml of penicillin–streptomycin. HepG2, Hep3B and PANC-1 cells were maintained in DMEM (Thermo Fisher) with 10% FBS and 100 mg/ml of penicillin-streptomycin. PLC/PRF/5 cells were maintained in MEM-α (Thermo Fisher) with 10% FBS and 100 mg/ml of penicillin-streptomycin. Cells were grown in a humidified atmosphere of 5% CO_2_ and 95% air at 37^°^C. The following reagents were used: TGFβ-1 (Peprotech, Rocky Hill, NJ, USA); TGFβ receptor I-specific inhibitor 616451 (EMD Millipore, Billerica, MA, USA); SMAD3-specific inhibitor SIS3 (Sigma-Aldrich, St. Louis, MO, USA); Scriptaid, Trichostatin A, and 5-fluorouracil were from Sigma-Aldrich.

### Plasmids

pCMV-p53-R175H, p53-R248Q, p53-R249S, p53-R273H, p53-R280K, p53-D281G were generated as previously described [21]. pcDNA3.1-p300 and pcDNA3.1-p300(HAT-) were from Warner Greene (Addgene #23252 and #23254). The pGL2-p21 promoter-Luc was from Martin Walsh (Addgene #33021). The NOX4-pGL3 luciferase reporter plasmids -4760 and -3975 were provided by Victor Thannickal (University of Alabama) [22]. Other NOX4-pGL3 promoter reporter plasmids were generated as previously described [35]. The pGL3-Basic control vector was from Promega (Promega Corporation, Madison, WI, USA). TGFβRI (T204D) was previously described [34]. SMAD2 and SMAD3 were from Lalage Wakefield (NIH/NCI, Bethesda, MD). Active SMAD2 and SMAD3 were pseudo-phosphorylated at serine residues (SMAD2: serine residues 464, 465, and 467) (SMAD3: serine residues 422, 423, and 425) by replacement with aspartic acid residues.

### Transient transfections

Hep3B, PCL/PRF/5, PANC-1, and H1299 were transfected with Fugene 6 (Promega, Madison, WI, USA) using manufacturer's protocols. H1299 cells, for migration, studies were transfected with Lipofectamine 3000 (Thermo Fisher) according to the manufacturer's protocols.

### siRNA-mediated gene knockdown

Dharmacon ON-TARGETplus SMARTpool siRNAs were used for silencing endogenous p53 (TP53 siRNAs L-003329-00-0005) and p300 (EP300 siRNAs L-003486-00-0005) (Dharmacon, Lafayette, CO, USA). Non-targeting siRNAs (D-001810-01-05) were used as the control. DharmaFECT4 reagent was used for siRNA transfections according to manufacturer's protocols. The cells were transfected in antibiotic-free medium at a concentration of 50 nM for 72 hours.

### RNA isolation and cDNA synthesis

Total RNA from cells was extracted with Trizol (Thermo Fisher). One microgram of total RNA was used for Thermoscript (Thermo Fisher) RT-PCR. Both were conducted according to manufacturer's protocols.

### Quantitative PCR

Gene expression was quantified by qPCR using an ABI Prism 7500 RT-PCR System (Applied Biosystems, Foster City, CA, USA). Cellular RNA was reverse transcribed with ThermoScript. SYBR Green PCR mix (Thermo Fisher) was used to detect mRNA expression with the following human specific primers: (NOX4) forward: 5′-TGAACTATGACCTCAGCCTCTGCG-3′, reverse: 5′-ATGACTGGAAACCATACAAGCT-3, (p21/CDKN1A) forward: 5′-CCGAAGTCAGTTCCTTGTGG-3′, reverse: 5′-CATGGGTTCTGACGGACAT -3′ and (GAPDH) forward: 5′-AGCCACATCGCTCAGACAC-3′, reverse: 5′-GCCCAATACGACCAAATCC-3′. GAPDH was used as an internal control for normalization. The qPCR results are represented as relative quantification (RQ), which is RQ = 2^(−ΔΔCt)^. The RQ value is the fold change in gene expression compared to the control sample.

### Luciferase assay

Luciferase assays were performed according to manufacturer's protocols (Promega). Briefly, 1 × 10^5^ cells were seeded per well in 12-well dishes. Each well was transfected with 0.5 μg of pGL3-NOX4 promoter reporter plasmid 24 hours later. Luciferase expression was assayed 48 hours later with Luminoskan luminometer (Thermo Fisher).

### Chromatin immunoprecipitation (ChIP)

Cells were cultured on 150 mm and fixed with 1% formaldehyde (10 min, RT), followed by 200 mM Glycine for 10 min. The cells were washed twice with ice-cold 1 X PBS, and collected in lysis buffer (1 × PBS containing protease inhibitor cocktail (PIC) and 0.5 mM PMSF). Chromatin preparations were sheared using ChIP-IT Express Enzymatic Shearing Kit according to manufacturer's protocols (Active Motif, Carlsbad, CA, USA). Immunoprecipitations were performed using Magna ChIP A/G Kit according to manufacturer's protocol (Millipore). The following antibodies were used for immunoprecipitation: anti-SMAD3 (E.980.9) (Thermo Fisher); anti-acetyl histone H4 (Lys8) (Millipore); anti-p53 (DO-1); mouse IgG (Santa Cruz, Dallas, TX, USA). Quantitative PCR was used to quantify the immunoprecipitated DNA with the following NOX4 promoter-specific primers: (R1) forward: 5′- AAGGGCATAAGGACCTCTCC-3′, reverse: 5′- AGGGAAAAGTGGTCCAAAG-3′; (R2) forward: 5′- CTGAATCAGATGATGGTCTACACTTG-3′, reverse: 5′- GGTCCAAAGGCTTAACATTC-3′; (R3) forward: 5′- AAGGGCATAAGGACCTCTCC-3′, reverse: 5′- GACTCATTCTCATTTCTAC-3′. The data are represented as fold change over IgG control antibody (Ct IP/Ct IgG). The Ct values were normalized to the input (1%) of sheared chromatin DNA. The data are represented as fold change above background = 2^(^−ΔΔCt^).

### Western blotting

Cell lysates were processed for Western blotting as previously described [21]. The following antibodies were used for immunoblotting: rabbit monoclonal anti-NOX4 (UOTR1B493) (Abcam, Cambridge, MA, USA); mouse monoclonal anti-p53 (DO-1) (Santa Cruz Biotechnology); rabbit monoclonal anti-phospho-SMAD3 (EP823Y) (Abcam); rabbit monoclonal anti-SMAD3 (EP568Y) (Abcam); and rabbit polyclonal anti-p300 (A300-358A) (Bethyl Laboratories, Montgomery, TX, USA).

### Cell migration assay

H1299 cells were seeded on 6-well tissue culture plates (4 × 10^5^ cells/ well) 24 hours before transfection. The cells were transfected with 2 μg of vector control, p53-WT, p53-D281G, p300-WT, p300-ΔHAT, or NOX4-P437H plasmid DNA for 5 hours. The cells were then trypsinized and re-seeded into 96-well tissue culture plates (3.5 × 10^4^ per well) for monolayer wound-healing assays. Wounds were made using WoundMaker (Essen Bioscience, Ann Arbor, MI, USA) 96-pin tool, creating reproducible uniform cell-free zones of 800 μm. Immediately following wounding, cells were washed twice then treated with TGFβ (5 ng/ml) or left untreated. Approximately 18-22 hours after wounding and TGFβ treatment, the cells were fixed and stained using Diff Stain (IMEB Inc., San Marcos, CA, USA). Visible light images (4 × objective) of fixed and stained migrating cells were captured with a phase contrast microscope (Evos FL Cell Imaging System, Life Technologies, Carlsbad, CA, USA).

### Statistical analysis

Data are represented as means ± s.d. of the results of at least three independent experiments. Student's *t*-test was used to calculate significant values, indicated as *P-value < 0.05, **P-value < 0.01, or ***P-value < 0.001.

## Abbreviations

TGFβ: Transforming growth factor-β
NOX4: NADPH oxidase-4
ROS: Reactive oxygen species
HAT: Histone acetyltransferase
HDAC: Histone deacetylase
SBE: SMAD binding element
p53RE: p53 response element
